# Use of secondary prevention pharmacotherapy after first myocardial infarction in patients with diabetes mellitus

**DOI:** 10.1186/1471-2261-14-4

**Published:** 2014-01-09

**Authors:** Casper H Jørgensen, Gunnar H Gislason, Ole Ahlehoff, Charlotte Andersson, Christian Torp-Pedersen, Peter R Hansen

**Affiliations:** 1 Department of Cardiology, Copenhagen University Hospital Gentofte, Niels Andersens Vej 65, Copenhagen 2900, Denmark; 2 Department of Health Science and Technology, Aalborg University, Niels Jernes Vej 12, Aalborg 9220, Denmark

## Abstract

**Background:**

Despite recommended pharmacotherapies the use of secondary prevention therapy after myocardial infarction (MI) remains suboptimal. Patients with diabetes mellitus (DM) have worse prognosis after MI compared to patients without DM and aggressive secondary prevention pharmacotherapy in this population is therefore warranted. We examined the changes in use of evidence-based secondary prevention pharmacotherapy in patients with and without DM discharged after first MI.

**Methods:**

All patients aged 30 years or older admitted with first MI in Denmark during 1997–2006 were identified by individual-level linkage of nationwide registries of hospitalizations. Univariate and multivariate logistic regression models were used to identify patient characteristics associated with initiation of acetylsalicylic acid, angiotensin-converting enzyme inhibitors or angiotensin receptor blockers, β-blockers, and clopidogrel within 90 days, and statins within 180 days of discharge, respectively.

**Results:**

A total of 78,230 patients were included, the mean age was 68.3 years (SD 13.0), 63.5% were men and 9,797 (12.5%) had diabetes. Comparison of claimed prescriptions in the period 1997–2002 and 2003–2006 showed significant (p < 0.001) increases in claims for acetylsalicylic acid (38.9% vs. 69.7%), angiotensin-converting enzyme inhibitors or angiotensin receptor blockers (38.7% vs. 50.4%), β-blockers (69.2% vs. 77.9%), clopidogrel (16.7% vs. 66.3%), and statins (41.3% vs. 77.3%). During 2003–2006, patients with DM claimed significantly less acetylsalicylic acid (odds ratio [OR] 0.81 [95% confidence interval [CI] 0.74–0.88) and clopidogrel (OR 0.91 [95% CI 0.83–1.00]) than patients without DM.

**Conclusions:**

Despite sizeable increase in use of evidence-based secondary prevention pharmacotherapy after MI from 1997 to 2006, these drugs are not used in a substantial proportion of subjects and patients with DM received significantly less antiplatelet therapy than patients without DM. Increased focus on initiation of secondary prevention pharmacotherapy after MI is warranted, especially in patients with DM.

## Background

Optimal use of evidence-based secondary prevention pharmacotherapy in patients with myocardial infarction (MI) reduces the risk of subsequent cardiovascular events and mortality [1-5]. The internationally recommended pharmacotherapies based on confirmative trials for secondary prevention after MI during the study period 1997–2006 included treatment with platelet inhibitors (acetylsalicylic acid [ASA] and a thienopyridine, e.g. clopidogrel), β-blockers, and lipid-lowering agents for most patients without contraindications and irrespective of reperfusion therapy [6-12]. Furthermore, high-risk post-MI patients with diabetes mellitus (DM), clinical heart failure, and/or left ventricular dysfunction should additionally receive angiotensin-converting enzyme inhibitors/angiotensin 2 receptor blockers (ACEIs/ARBs) [5,13].

Patients with DM have worse prognosis after MI compared to patients without DM and aggressive secondary prevention pharmacotherapy is warranted in DM patients [14]. Despite substantial data, however, the use of secondary prevention therapy after MI remains suboptimal [15]. To further examine this clinically important topic, the current study used population-based administrative databases to examine the temporal development in use of evidence-based secondary prevention pharmacotherapy in post-MI patients, with particular focus on patients with DM.

## Methods

### Health care system in Denmark

All permanent residents in Denmark can use the Danish health care system freely and are entitled to free treatment at a hospital. Data in the present study were obtained from admissions all of 82 existing public hospitals in Denmark in 1996. Expenses for the cost of drugs are partially reimbursed. The more expenses patients have for reimbursable drugs, the more reimbursement they will receive. Annual medical expenses >900 Danish Kroners (DKr) are reimbursed by 50%, >1,470 DKr by 75%, and >3,180 DKr by 85%. Moreover, general practitioners can apply (usually successfully) for 100% reimbursement of drug expenses payable to their patients with chronic conditions, i.e. DM.

#### Databases

In Denmark all citizens have a unique personal civil registration number, allowing individual linkage of information across nationwide registers. The Danish National Patient Register holds information of all admissions and invasive therapeutic procedures performed in Danish hospitals since 1978. Each admission is registered by one primary diagnosis and, if appropriate, one or more secondary diagnoses, coded according to the 8^th^ or 10^th^ revision of the International Classification of Diseases (ICD-8 code 410 for 1978–94 and ICD-10 codes I21–I22 from 1995 and onwards). The diagnosis of MI has previously been validated in the National Patient Registry with a specificity of 93% [16]. The Danish Register of Medicinal Product Statistics (National Prescription Register) holds information on all prescriptions dispensed from Danish pharmacies since 1995. Drugs are registered according to the international Anatomical Therapeutical Chemical (ATC) classification system. The national health care reimbursement scheme of drug expenses requires pharmacies in Denmark to register all dispensed prescriptions, which ensures complete registration [17]. Percutaneous coronary interventions (PCI) and coronary by-pass grafting (CABG) procedures performed <30 days after admission were detected by use of the Danish Health Care Classification System using the codes KFNG and KNFA-KNFE, respectively.

#### Study population

Patients aged ≥30 years admitted to Danish hospitals during 1997–2006 with a diagnosis of first MI (ICD-10 codes I21–I22) were identified from the Danish National Patient Registry. To certify the first hospitalization for MI, we traced the hospitalizations of all patients back to 1978. Patients with DM were identified in the Danish Register of Medicinal Product Statistics as individuals claiming at least one prescription for glucose-lowering drugs (GLDs; ATC A10), including all oral agents and insulin, in the period from 180 days before to 90 days after admission for MI. Although use of GLDs represents a conservative approach to identification of DM, this strategy has been shown to capture at least 85% of patients with DM in Denmark and it has a positive predictive value of 98% [18]. In 2002 the treatment strategy for ST-segment elevation myocardial infarction (STEMI) in Denmark was changed from fibrinolysis to primary PCI after results of the DANAMI II trial were presented [19]. Based on these treatment changes in 2002, we therefore specifically compared patients with first MI in the period 1997–2002 with patients admitted in 2003–2006.

#### Pharmacotherapy

We identified secondary prevention pharmacotherapy prescriptions claimed ≤90 days of discharge after first MI. The following drugs (ATC codes) were identified: ASA (B01AC06), ACEIs/ARBs (C09), β-blockers (C07), clopidogrel (B01AC04), and statins (A10A). Initiation of statin treatment has been shown to increase during the first 6 months after discharge after MI [20] and therefore prescription claims within the first 180 days after discharge were selected to identify patients that initiated statin therapy. Initiation of β-blockers and ACEIs/ARBs has been shown to occur primarily during the first 30 days after MI [20]. In addition, claims of loop-diuretics (C03C) and proton pump inhibitors (PPIs; A02B) were identified as proxies for heart failure (HF) and dyspeptic disease, respectively.

#### Comorbidity

Comorbidity was defined according to the modified Ontario Myocardial Infarction Mortality Prediction Rules by admission diagnoses at the index admission and 1 year previously [21]. The following diagnoses (ICD-10 codes) were used: HF (I42, I43, I50) cardiac dysrhythmias (I47-I49), pulmonary edema (J81), shock (R57), cerebrovascular disease (I60-I69), DM with chronic complications (E10-E14), acute renal failure (N17, N19, R34), chronic renal failure (N18, I12, I13) and malignancy (C00-C97).

#### Statistical analyses

All continuous variables were described as mean values ± SD. Comparisons between groups were made using unpaired *t-*test and χ^2^ test for discrete variables. Multivariable logistic regression models were used to analyze differences in odds ratios (ORs) for drug use between patients with and without DM, women and men, different patient age groups and between the two study periods, respectively. Patients were stratified in 5 age groups, i.e., 30–49 years, 50–59 years, 60–69 years, 70–79 years, and ≥80 years, and in age-dependent analyses, patients aged 60–69 years were chosen as the reference. Analyses were adjusted for comorbidity and concomitant pharmacotherapy. The linearity of continuous variables and lack of interactions was tested and found valid unless otherwise reported. Statistical calculations were performed with the SAS statistical software package, version 9.1 (SAS Institute Inc., Cary, NC).

#### Ethics

The Danish Data Protection Agency approved the study (No. 2007-41-1667). Data were delivered with anonymized, unique personal identification numbers, enabling linkage between registers on an individual level. Retrospective register studies where individuals cannot be identified do not require ethical approval in Denmark [22].

## Results

**Figure 1:**
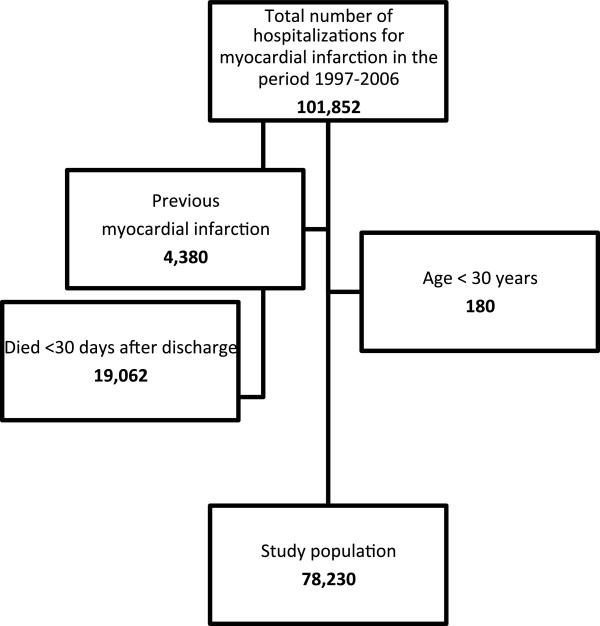
The selection of patients aged ≥30 years and alive 30 days after first myocardial infarction admitted to a Danish hospital during 1 January 1997–31 December 2006.

During the 10-year study period, 101,852 patients with MI were identified. Of these, 78,230 patients ≥30 years of age alive 30 days after discharge after their first MI were included in the study. The selection of the study population is shown in Figure 1. The study population comprised 49,665 (63.5%) men with a mean age of 65.7 years (SD 12.6), and 9,797 (12.5%) that claimed GLDs in the period 180 days before or 90 days after the index admission were identified as the study DM population. The prevalence of DM increased from 12.1% in 1997–2002 to 13.1% in 2003–2006. During the 30 to 90 days after discharge 3,951 (5.1%) died of which 628 (15.9%) had DM, leaving 74,279 patients to claim drug prescriptions for the entire period after discharge. For the entire period, patients with DM accounted for 12.3% of those surviving ≥ 90days. Patients with DM were older, i.e., 69.2 (SD 11.8) years vs. 68.1 (SD 13.2) years, more often women (39.4% vs. 36.1%; p < 0.0001) and had more comorbidity at baseline, especially HF (27.5% vs. 17.4%; p < 0.0001), cardiac dysrhythmias (11.1% vs. 10.2%; p < 0.0001), and cerebrovascular disease (7.2% vs. 4.4%; p < 0.0001), compared to patients without DM. The use of loop diuretics (a proxy for HF) in the late study period was significantly higher in DM patients than in patients without DM (52.7% vs. 32.4%; p < 0.0001). Women claimed more loop diuretics (OR 1.19 [95% CI 1.15-1.21]) than men. These findings were consistent for both study periods (1997–2002 and 2003–2006) and were independent of DM status. Baseline characteristics of the study population with and without DM are shown in Table 1.

**Table 1:** Baseline characteristics

	**No DM**	**DM**	**p-value**
**Number:**	**68,433**	**9,797**	
**Percentage of study population:**	87.5	12.5	
Gender, female (%)	36.1	39.4	<0.0001
Age at hospitalization (years ± SD )	68.1 (±13.2)	69.2 (±11.8)	<0.0001
**Period of hospitalization (%)**			
1997–2002	87.9	12.1	
2003–2006	86.9	13.1	
**Secondary prevention claimed 1997–2002 (%)**	**40,982**	**5,649**	
ASA	39.1	37.4	<0.0149
ACEIs/ARBs	36.4	55.1	<0.0001
β-blockers	69.8	64.8	<0.0001
Clopidogrel	17.0	14.1	<0.0001
Statins	41.6	39.1	<0.0003
**Secondary prevention claimed 2003–2006 (%)**	**27,451**	**4,148**	
ASA	70.5	64.3	<0.0001
ACEIs/ARBs	48.0	66.3	<0.0001
β-blockers	78.2	75.8	<0.0005
Clopidogrel	66.9	62.2	<0.0001
Statins	77.3	77.5	0.8050
**Comorbidity diseases (%)**			
Cardiac dysrhythmias	10.2	11.1	<0.0038
Heart failure	17.4	27.5	<0.0001
Pulmonary edema	1.0	1.8	<0.0001
Shock	0.9	1.5	<0.0001
Cerebrovascular disease	4.4	7.2	<0.0001
Renal disease	1.5	3.5	<0.0001
Diabetes complications	0.5	33.1	<0.0001
Malignancy	0.5	0.5	0.2953
**Concomitant pharmacotherapy**			
Loop diuretics	35.1	54.4	<0.0001
Proton pump inhibitors	18.1	21.2	<0.0001
**Reperfusion therapy**			
Percutaneous coronary intervention	28.0	21.8	<0.0001
Coronary artery by-pass graft	4.8	5.9	<0.0001

DM: diabetes mellitus, SD: standard deviation, ASA: acetyl salicylic acid, ACEI: angiotensin-converting enzyme inhibitor, ARB: angiotensin receptor blocker, CI: confidence interval.

### Initiation of secondary prevention pharmacotherapy

**Figure 2:**
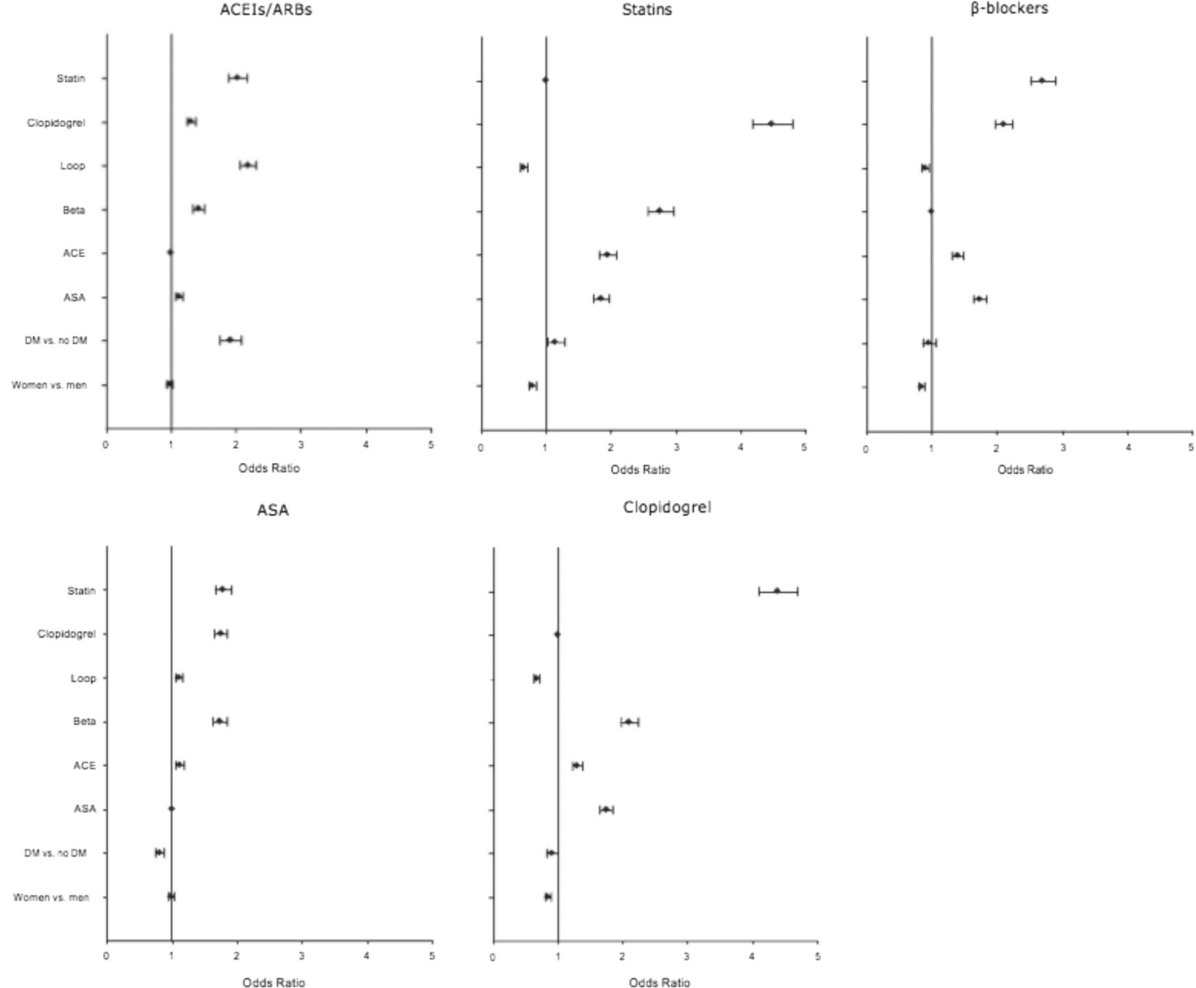
**Multivariable logistic regression analyses showing odds ratios for claiming secondary prevention therapy after first myocardial infarction during 2003–2006 for acetylsalicylic acid (ASA), angiotensin-converting enzyme inhibitors or angiotensin-2 receptor blockers (ACEIs/ARBs), β-blockers, clopidogrel, and statins.** DM: diabetes mellitus. Analyses were adjusted for age, gender, comorbidity, and concomitant pharmacotherapy.

The proportion of patients that claimed secondary prevention therapy increased significantly over time, i.e., for patients with DM the following increase in drug use between 1997–2002 and 2003–2006 was observed: ASA from 37.4% to 64.3%, ACEIs/ARBs from 55.1% to 66.3%, β-blockers from 64.8% to 75.8%, clopidogrel from 14.1% to 62.2%, and statins from 39.1% to 77.5% (all p<0.0001). Similarly for patients without DM drug use increased as follows: ASA from 39.1% to 70.5%, ACEIs/ARBs from 36.4% to 48.0%, β-blockers from 69.8% to 78.2%, clopidogrel from 17.0% to 69.9%, and statins from 41.6% to 77.3% (all p < 0.001). During the entire study period 1997–2006 patients with DM claimed significantly less evidence-based secondary prevention pharmacotherapy compared with patients without DM, except for ACEIs/ARBs and statins in the late (2003–2006) study period. An unadjusted analysis showed that the ORs for receiving ASA, β-blockers, and clopidogrel were significantly lower in patients with DM compared to patients without DM, while OR for treatment with ACEIs/ARBs was increased in patients with DM (Table 2). Figure 2 shows multivariable logistic regression analyses with ORs for claims of secondary prevention pharmacotherapy in the period 2003–2006 after adjustments for age, gender, and comorbidity. In 2003–2006 patients with DM claimed less ASA (OR 0.81 [95% CI 0.74-0.88]) and clopidogrel (OR 0.91 [95% CI 0.83-1.00]) than patients without DM (Figure 2). Sensitivity analyses excluding patients with vs. without DM claiming ASA (26.5% vs. 15.0%) and clopidogrel (1.5% vs. 2.2%) 90 days prior to admission provided similar results and in this analysis patients with DM claimed less ASA (OR 0.76 [95% CI 0.69-0.84]) and clopidogrel (OR 0.90 [95% CI 0.82-0.98]) than patients without DM. Statistically significant interactions were found between all examined secondary prevention pharmacotherapy agents and patient gender and age. The ORs for claiming secondary prevention therapy were found to be significantly lower in women than in men for β-blockers (OR 0.84 [95% CI 0.79–0.89]), clopidogrel (OR 0.84 [95% CI 0.80–0.89]), and statins (OR 0.79 [95% CI 0.74–0.84]). The proportion of patients that claimed secondary prevention therapy decreased significantly with increasing age, except for claims of ASA, and this age-dependent decrease in secondary prevention therapy was most apparent for β-blockers and statins, i.e., patients aged >69 years had OR 0.86 (95% CI 0.79–0.93) for receiving β-blockers and OR 0.63 (95% CI 0.57–0.70) for receiving statins, compared to patients aged 60–69 years, respectively. Furthermore, as shown in Table 1, patients with DM underwent significantly fewer PCI procedures and significantly more CABG procedures (both p < 0.0001).

**Table 2:** Univariate analysis

	**OR**	**95% CI**	***p***
**ASA**	0.75	0.70–0.81	<0.0001
**ACEIs/ARBs**	2.13	1.99–2.28	<0.0001
**β-blockers**	0.87	0.80–0.94	<0.0001
**Statins**	1.01	0.93–1.09	0.8054
**Clopidogrel**	0.81	0.76–0.87	<0.0001

Odds ratio (OR) for claiming secondary prevention pharmacotherapy after first myocardial infarction during 2003–2006 in patients with vs. without diabetes mellitus. ASA: acetyl salicylic acid, ACEI: angiotensin-converting enzyme inhibitor, ARB: angiotensin receptor blocker, CI: confidence interval.

### Combinations of secondary prevention pharmacotherapy agents

**Figure 3:**
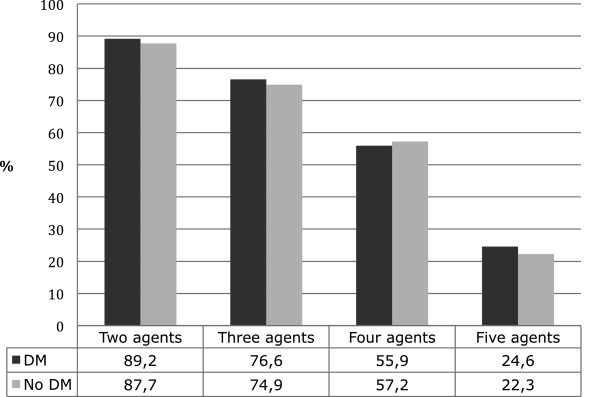
**Use of secondary prevention drugs after first myocardial infarction in combinations of two, three, four or five agents including acetylsalicylic acid, angiotensin-converting enzyme inhibitors or angiotensin-2 receptor blockers, β-blockers, clopidogrel, and statins, respectively, claimed ≤90 days after discharge during the period 2003–2006.** DM: diabetes mellitus.

The ORs for claiming two or more secondary prevention drugs increased markedly between the two study periods. In the total study population the proportion of patients claiming a combination of 2, 3, 4 and 5 agents increased from 62.3% to 87.9%, 34.4% to 75.1%, 12.9% to 57.1% and 2.8% to 22.6 respectively, between 1997–2002 and 2003–2006. Patients with DM claimed more ACEIs/ARBs in any combination than patients without DM. A comparison of the use combinations of 2, 3, 4 and 5 agents, for patients with and without DM during 2003–2006, is shown in Figure 3.

## Discussion

In this nationwide study of patients discharged after their first MI in Denmark, we found that the proportion of patients that claimed secondary prevention pharmacotherapies increased substantially from 1997–2002 to 2003–2006. Patients with DM, however, claimed less ASA and clopidogrel than patients without DM, and women and the elderly generally received less secondary prevention agents. In the total study population, the fraction of patients claiming a combination of two or more secondary prevention pharmacotherapy agents increased significantly during the study period but combination therapies appear to remain underused as compared to treatment guidelines [2,3,23]. Although previous studies have shown that even in the modern era patients with coronary artery disease generally receive suboptimal secondary prevention pharmacotherapy [15,24], our study adds important information on treatment differences between patients with and without DM, genders, and age groups, respectively, in an unselected nationwide post-MI population. Specifically, the data suggest that in post-MI patients with DM, there should be increased focus on increased provision of antiplatelet therapy with ASA and clopidogrel and of combinations of evidence-based secondary prevention drug therapies. In the general population, there also remains considerable room for improvement of secondary prevention pharmacotherapy, particularly in women and the elderly.

### Antiplatelets

Platelet inhibitors, i.e., low dose ASA and clopidogrel are recommended for secondary prevention after MI irrespective of, for example, patient gender, age, and DM status [25-27]. In the current study, post MI patients with DM claimed significantly less ASA and clopidogrel than patients without DM. The reasons for this under-treatment are not readily apparent but patients with DM underwent more CABG than patients without DM (Table 1), and contrary to treatment guidelines, surgeons may discontinue clopidogrel after CABG in post MI patients [28]. Patients ≥70 years of age claimed significantly less clopidogrel than the younger population, whereas the use of ASA was not affected by increasing age. Results from the Can Rapid risk stratification of Unstable angina patients Suppress ADverse outcomes with Early implementation of the American College of Cardiology/American Heart Association Guidelines (CRUSADE) initiative have showed that even very old (≥90 years) patients with acute coronary syndromes benefit from clopidogrel treatment [29]. Consistent with our findings, the use of ASA in patients with atherothrombotic disease was recently found to be lower in patients with DM than patients without DM in the international Reduction of Atherothrombosis for Continued Health (REACH) registry [30]. Patients with DM may have received less ASA due to clinicians’ concern of retinal bleeding in patients with retinopathy. However, the Early Treatment Diabetic Retinopathy Study Report 14 results from 1992 support treatment with ASA in patients with diabetes at increased risk of cardiovascular disease and found no increased risk of retinal bleeding associated with the use [6]. Female gender has previously been associated with lower use of ASA [29], but in or study there were no significant differences in claims of ASA prescriptions between women and men. Unsurprisingly, we found that a greater proportion of patients with DM compared to patients without DM claimed ASA and clopidogrel prior to admission, probably reflecting, in part, the increased baseline rate of cerebrovascular disease. The possibility that decreased utilization of ASA and clopidogrel after first MI in patients with DM was caused by these patients using their remaining stocks of the antiplatelet agents after discharge was not supported by our comparable finding in a sensitivity analysis that excluded patients with prior use of ASA and clopidogrel. Patients with DM have increased prevalence of gastrointestinal discomfort, e.g., due to gastroparesis, which may reduce initiation of and compliance with pharmacologic treatment, especially ASA and clopidogrel [31]. After including claims of PPIs 90 days after discharge in the multivariable analyses, however, there remained a significant underuse in DM compared to non-DM patients. Mortality rates 30–90 days after MI, i.e., the period where prescription claims were retrieved according to our study design, did not differ significantly between patients with and without DM and are therefore unlikely to have contributed to the observed discrepancy in drug use between the two groups.

### β-blockers

Secondary prevention with β-blockers after MI are recommended for all patients and is particularly important because immediate treatment can reduce mortality and risk of re-MI [2]. The absolute benefit of a given relative reduction of post-MI risk may be greater in patients with DM because of their higher absolute risk. In the current study, the univariate analysis demonstrated a significantly lower β-blocker use in DM patients (Table 2) and the multivariate analysis (Figure 2) showed a similar trend. Since β-blockers are also a mainstay in treatment of severe HF, this apparent under treatment is reinforced by the increased prevalence of HF in DM patients after MI during the late (2003–2006) study period. Among the reasons contributing to this finding may be the widespread misconception that β-blockers may mask symptoms of hypoglycemia and prolong recovery from hypoglycaemia [32], although the value of β-blocker is post MI DM patients has been firmly established [33]. Also, patients with DM in our study were slightly older than their non DM controls and the elderly are at an increased risk of adverse cardiac effects of β-blockers, e.g., low cardiac output and bradycardia, which together with their increased prevalence of chronic obstructive lung disease may have added to the underuse of β-blockers in patients with DM.

### Angiotensin-converting enzyme inhibitors and angiotensin receptor blockers

The international guidelines generally recommend ACEI/ARB for secondary post MI prevention in patients with HF, left ventricular ejection fraction less than 45%, DM, hypertension, chronic renal disease, peripheral arterial disease, and/or when patients are otherwise considered as being at high risk [34,35]. In the current study, 50.4% of patients claimed ACEIs/ARBs, which is markedly lower than reported in the European Action on Secondary and Primary Prevention through Interventions to Reduce Events (EUROASPIRE) III survey where 71% reported use of these agents six months after an acute coronary syndrome [24]. Patients included in that study, however, were not exclusively those with first-time MI but also included subjects with a recurrent diagnosis of or treatment for coronary artery disease which could lead to more patients receiving treatment [24]. We found that patients with DM claimed significantly more ACEIs/ARBs than patients without DM, i,e., 66.3% vs. 48.0%, in agreement with the guideline recommendation, and the increased prevalence of HF and renal disease in these patients (Table 1) [36]. The risk of lactic acidosis relating to concomitant use of metformin in subjects with DM and renal impairment may also have contributed to the underuse of ARB/ACEI in patients with DM.

### Statins

During the study period, the increase in the number of patients claiming statins was greater than the increase in the use of any of the other secondary prevention agents. Indeed, 77.4% of patients claimed statins in 2003–2006, and the increase in statin use was independent of DM status. During the study period several landmark studies and guidelines on primary and secondary prevention of cardiovascular disease with statins were published which probably contributed to the increased use [37-39]. Based on current evidence all patients with MI and in particular patients with DM should receive statins [40]. Even a higher fraction of patients with DM claiming statins may therefore have been expected in the present study. The proportion of women claiming statins was consistently lower than the proportion of men, regardless of subject age. Women have lower primary risk of coronary artery disease than men, but in the post-MI population there should be no differences in secondary prevention statin use between genders. In particular, the elderly (≥70 years) population claimed significantly fewer prescriptions for statins, even though results of the Prospective Study of Pravastatin in the Elderly at Risk (PROSPER) study published in 2002 clearly showed a reduced risk of coronary death and non-fatal MI in a population aged 70–82 years with a history of (or risk factors for), vascular disease.

### Combinations of secondary prevention drugs

A combination of secondary prevention pharmacotherapies is frequently needed to optimize myocardial function post MI and prevent subsequent adverse events [25,35]. Based on the marked survival advantages associated with combination treatment of post MI patients the present results are disappointing [41]. In our study only 32.1% of patients with DM and 27.0% without DM claimed prescriptions for ASA, ACEIs/ARBs, β –blockers, and statins during the first 90 days post-MI. According to current evidence DM patients should receive more aggressive post MI therapy than patients without DM to reduce their increased risk and the fact that they received less combination therapy, apart from combinations containing ACEIs/ARBs, is disturbing. Our data essentially suggest a substantial underuse of combinations of recommended secondary prevention therapies after MI irrespective of DM status, not least since synergistic effects of co-administration of such therapies are reasonably well established. For example, in the Steno 2 trial published in 2003 [42] intensive multifactorial interventions including ACEIs/ARBs, β –blockers, and statins in patients with type 2 DM and microalbuminuria reduced the risk of cardiovascular and microvascular events by about 50%. The publication of this study may have increased awareness of clinicians of the effectiveness of both primary and secondary preventive therapies in patients with diabetes. Concerns about possible drug interactions leading to inadequate initiation of therapies and poor patient compliance in patients with chronic disease may also contribute to drug underuse [43].

Numerous previous studies have examined the proportion of hospitalized cardiac patients discharged with secondary prevention medications. A meta-analysis of data from 376,162 patients in 20 studies of primary and secondary prevention (9 studies) assessing adherence to aspirin, ACEIs, ARBs, *β*-blockers, calcium channel blockers, thiazides, and statins found a mean general adherence to secondary prevention therapies of 66% [44]. A registry study of a 2008–2009 population cohort in Spain of patients admitted with acute coronary syndrome found the proportions of drug adherence to be 69.9%, 45.4%, 43.3%, and 58.8%, for antiplatelet drugs, ACEIs/ARBs β-blockers and statins, respectively. In that study only 47.6% received 3 or more drugs and no association was found between low drug adherence and DM [45]. Although we did not specifically examine drug adherence but only used the correlate of claimed prescriptions the use of ACEIs/ARBs, *β*-blockers and statins in the late study period (2003–2006) in the present study is consistent with these more recent results albeit that the percentage of patients that used combinations of 3 or more drugs (75,1%) in our study was markedly higher.

### Methods to improve adherence to secondary prevention therapies

Adherence is a key factor associated with the effectiveness of all pharmacological therapies not least for medications prescribed for chronic conditions like cardiovascular diseases and DM. Medical adherence is a complex issue and involves demographic, psychological, and social factors, as well as factors related to the health care provider, medical system, specific disease and treatments. Improved adherence may be achieved by targeted education of patients, involving patients in decisions regarding their treatments, changes of dosing regimens, reduction of adverse effects, and improvements of access to medication and medical consultations.

### Limitations and strengths

The main study limitation is inherent in the observational study design. The registers did not include information on important confounders, e.g., left ventricular ejection fraction, renal function, and contraindications for treatment. Additionally, we did not know the number of patients who initiated secondary prevention therapy during hospitalization but did not tolerate treatment. Furthermore, the definition of DM was based on prescription claims for GLDs. Therefore DM patients treated with diet were identified as not having DM. The data collected in this study origin only from Denmark and the results should only be extrapolated to other countries with different health care systems with caution. The use of secondary prevention therapies after first MI according to the specific hospitals that patients were admitted to in Denmark has been investigated previously and was found to vary substantially [46]. In the present study, data from admissions to a total of 82 hospitals were included and clustering of prescription claims, e.g., with patients discharged from highly specialized cardiology clinics receiving more evidence-based drugs than those discharged from community hospitals, is likely to have influenced the results. Therefore, our results apply to an average of Danish hospitals and patients and cannot be extrapolated to represent the performance status of any specific institution. The data is now 5–15 years old but since the underlying fundamentals, e.g., the free-of-charge health care system and well-structured organisation of post-MI treatment in Denmark, have not changed we sincerely believe that today’s situation in terms of suboptimal treatment of a considerable number of patients is unlikely to be markedly different from our 1997–2006 results. Indeed, in view of these favourable features of the Danish health care system it is likely that implementation of secondary prevention therapies after MI is even worse in other countries. Therefore, the overall message of our paper is likely to remain valid and the implicit call for a focused effort to increase use of evidence-based secondary prevention drugs is probably as relevant today as in 2006.

The main strength of this study was the nationwide consecutive patient registries, the contemporary data collection, the large sample size comprising approximately 78,000 patients, and the use of pharmacy dispensations and not prescriptions alone, which better reflected true as opposed to intended drug use. Selection bias arising from inclusion of only subgroups of patients or patients from selected hospitals, medical centers, or health care systems was avoided. Furthermore, the cohort comprised patients both in and out of the labor market. In Denmark, a government-financed health care system ensures practically equal access to health care for all inhabitants.

## Conclusions

In patients with first MI, the proportion of subjects who claimed secondary prevention pharmacotherapies increased significantly over time during 1997–2006. However, patients with DM claimed significantly less ASA and clopidogrel and suboptimal use of drug combinations was found. Moreover, women and elderly patients were less treated with secondary prevention pharmacotherapy compared to men and younger individuals, respectively. A focused effort to increase use of evidence-based secondary prevention drugs in post MI patients with and without DM is likely to provide long-term benefit.

## Competing interest

The authors declare that they have no competing interest.

## Authors’ contributions

CJ, GG, CTP and PRH conceived and designed the study. CJ, OA, CA, PRH and GG acquired and analyzed data. CJ drafted the first version of the manuscript. All authors interpreted data and critically revised the manuscript for important intellectual content and accepted the final version of the manuscript for submission.

## Pre-publication history

The pre-publication history for this paper can be accessed here:

http://www.biomedcentral.com/1471-2261/14/4/prepub

## References

[B1] Wald NJ Law MR A strategy to reduce cardiovascular disease by more than 80% BMJ 2003 326 7404 1419 10.1136/bmj.326.7404.1419 12829553 PMC162259

[B2] Freemantle N Cleland J Young P Mason J Harrison J β Blockade after myocardial infarction: systematic review and meta regression analysis BMJ 1999 318 7200 1730 1737 10.1136/bmj.318.7200.1730 10381708 PMC31101

[B3] LaRosa JC He J Vupputuri S Effect of statins on risk of coronary disease: a meta-analysis of randomized controlled trials JAMA 1999 282 24 2340 2346 10.1001/jama.282.24.2340 10612322

[B4] Pfeffer MA McMurray JJV Velazquez EJ Rouleau J-L Køber L Maggioni AP Valsartan, captopril, or both in myocardial infarction complicated by heart failure, left ventricular dysfunction, or both N Engl J Med 2003 349 20 1893 1906 10.1056/NEJMoa032292 14610160

[B5] Mehta RH Eagle KA Secondary prevention in acute myocardial infarction BMJ 1998 316 7134 838 842 Epub 1998/04/29 9549457 10.1136/bmj.316.7134.838 PMC1112771

[B6] Aspirin effects on mortality and morbidity in patients with diabetes mellitus: early treatment diabetic retinopathy study report 14: ETDRS investigators JAMA 1992 268 10 1292 1300 Epub 1992/09/09 10.1001/jama.1992.03490100090033 1507375

[B7] Collaborative overview of randomised trials of antiplatelet therapy--I: prevention of death, myocardial infarction, and stroke by prolonged antiplatelet therapy in various categories of patients: antiplatelet trialists’ collaboration BMJ 1994 308 6921 81 106 Epub 1994/01/08 8298418 PMC2539220

[B8] Yusuf S Peto R Lewis J Collins R Sleight P Beta blockade during and after myocardial infarction: an overview of the randomized trials Progress in cardiovascular diseases 1985 27 5 335 371 Epub 1985/03/01 10.1016/S0033-0620(85)80003-7 2858114

[B9] Lancet A randomised, blinded, trial of clopidogrel versus aspirin in patients at risk of ischaemic events (CAPRIE): CAPRIE steering committee Lancet 1996 348 9038 1329 1339 Epub 1996/11/16 8918275 10.1016/s0140-6736(96)09457-3

[B10] Yusuf S Zhao F Mehta SR Chrolavicius S Tognoni G Fox KK Effects of clopidogrel in addition to aspirin in patients with acute coronary syndromes without ST-segment elevation N Engl J Med 2001 345 7 494 502 Epub 2001/08/25 11519503 10.1056/NEJMoa010746

[B11] Investigators THOPES Effects of an angiotensin-converting–enzyme inhibitor, ramipril, on cardiovascular events in high-risk patients N Engl J Med 2000 342 3 145 153 10639539 10.1056/NEJM200001203420301

[B12] Randomised trial of cholesterol lowering in 4444 patients with coronary heart disease: the Scandinavian Simvastatin Survival Study (4S) Randomised trial of cholesterol lowering in 4444 patients with coronary heart disease: the scandinavian simvastatin survival study (4S) Lancet 1994 344 8934 1383 1389 Epub 1994/11/19 7968073

[B13] Deedwania PC Amsterdam EA Vagelos RH Evidence-based, cost-effective risk stratification and management after myocardial infarction: California cardiology working group on post-MI management Archives of internal medicine 1997 157 3 273 280 Epub 1997/02/10 10.1001/archinte.1997.00440240027005 9040293

[B14] AmericanDiabetesAssociation Standards of medical care in diabetes - 2008 Diabetes Care 2008 31 1 S12 S54 18165335 10.2337/dc08-S012

[B15] Newby LK Allen LaPointe NM Chen AY Kramer JM Hammill BG DeLong ER Long-term adherence to evidence-based secondary prevention therapies in coronary artery disease Circulation 2006 113 2 203 212 10.1161/CIRCULATIONAHA.105.505636 16401776

[B16] Madsen M Davidsen M Rasmussen S Abildstrom SZ Osler M The validity of the diagnosis of acute myocardial infarction in routine statistics: a comparison of mortality and hospital discharge data with the Danish MONICA registry J Clin Epidemiol 2003 56 2 124 130 10.1016/S0895-4356(02)00591-7 12654406

[B17] Gaist D Sorensen HT Hallas J The Danish prescription registries Dan Med Bull 1997 44 4 445 448 9377907

[B18] Drivsholm TB Frederiksen K Olivarius NF Odegaard B Kristensen JK The prevalence of diabetes in Denmark: development of a method for a registry-based assessment Ugeskrift for laeger 2003 165 2887 2891 12908359

[B19] Andersen HR Nielsen TT Rasmussen K Thuesen L Kelbaek H Thayssen P A comparison of coronary angioplasty with fibrinolytic therapy in acute myocardial infarction N Engl J Med 2003 349 8 733 742 10.1056/NEJMoa025142 12930925

[B20] Gislason GH Rasmussen JN Abildstrøm SZ Gadsbøll N Buch P Friberg J Long-term compliance with beta-blockers, angiotensin-converting enzyme inhibitors, and statins after acute myocardial infarction Eur Heart J 2006 27 10 1153 1158 16399775 10.1093/eurheartj/ehi705

[B21] Tu JV Austin PC Walld R Roos L Agras J McDonald KM Development and validation of the ontario acute myocardial infarction mortality prediction rules J Am Coll Cardiol 2001 37 4 992 997 10.1016/S0735-1097(01)01109-3 11263626

[B22] Daasnes C The personal data act, clinical trials and data privacy: rules for treatment of personal data in clinical trials and scientific research projects Ugeskrift for laeger 2003 165 16 1683 1685 Epub 2003/05/22. Persondataloven, laegemiddelforsog og datasikkerhed. Reglerne for behandling af personoplysninger i laegemiddelforsog og laegevidenskabelige forskningsprojekter 12756831

[B23] Fox KM EURopean trial On reduction of cardiac events with Perindopril in stable coronary Artery disease Investigators Efficacy of perindopril in reduction of cardiovascular events among patients with stable coronary artery disease: randomised, double-blind, placebo-controlled, multicentre trial (the EUROPA study) Lancet 2003 362 9386 782 788 13678872 10.1016/s0140-6736(03)14286-9

[B24] Kotseva K Wood D De Backer G De Bacquer D Pyorala K Keil U EUROASPIRE III: a survey on the lifestyle, risk factors and use of cardioprotective drug therapies in coronary patients from 22 European countries Eur J Cardiovasc Prev Rehabil 2009 16 2 121 137 10.1097/HJR.0b013e3283294b1d 19287307

[B25] Ryden L Standl E Bartnik M Van den Berghe G Betteridge J de Boer M-J Guidelines on diabetes, pre-diabetes, and cardiovascular diseases: executive summary Eur Heart J 2007 28 1 88 136 17220161 10.1093/eurheartj/ehl260

[B26] Antithrombotic TC Aspirin in the primary and secondary prevention of vascular disease: collaborative meta-analysis of individual participant data from randomised trials Lancet 2009 373 9678 1849 1860 19482214 10.1016/S0140-6736(09)60503-1 PMC2715005

[B27] Yusuf S Zhao F Mehta SR Chrolavicius S Tognoni G Fox KK Clopidogrel in Unstable Angina to Prevent Recurrent Events Trial Investigators Effects of clopidogrel in addition to aspirin in patients with acute coronary syndromes without ST-segment elevation N Eng J Med 2001 345 7 494 502 10.1056/NEJMoa010746 11519503

[B28] Sørensen R Abildstrøm SZ Hansen PR Hvelplund A Andersson C Charlot M Efficacy of post-operative clopidogrel treatment in patients revascularized with coronary artery bypass grafting after myocardial infarction J Am Coll Cardiol 2011 57 10 1202 1209 10.1016/j.jacc.2010.09.069 21371637

[B29] Adam HS Karen PA Anita YC Matthew TR Charles VP Ohman EM Characteristics, management, and outcomes of 5,557 patients age ≥90 years with acute coronary syndromes: results from the CRUSADE initiative J Am Coll Cardiol 2007 49 17 1790 1797 10.1016/j.jacc.2007.01.066 17466230

[B30] Cannon CP Rhee KE Califf RM Boden WE Hirsch AT Alberts MJ Current use of aspirin and antithrombotic agents in the united states among outpatients with atherothrombotic disease (from the REduction of atherothrombosis for continued health [REACH] registry) Am J Cardiol 2010 105 4 445 452 10.1016/j.amjcard.2009.10.014 20152237

[B31] Ma J Rayner CK Jones KL Horowitz M Diabetic gastroparesis: diagnosis and management Drugs 2009 69 8 971 986 10.2165/00003495-200969080-00003 19496627

[B32] Lang N Fox K Current drug therapies for the secondary prevention of MI Prescriber 2008 19 1 14 25 10.1002/psb.173

[B33] UK Prospective Diabetes Study Group Efficacy of atenolol and captopril in reducing risk of macrovascular and microvascular complications in type 2 diabetes: UKPDS 39 BMJ 1998 317 7160 713 720 10.1136/bmj.317.7160.713 9732338 PMC28660

[B34] Skinner JS Cooper A Feder GS Secondary prevention for patients following a myocardial infarction: summary of NICE guidance Heart 2007 93 7 862 864 10.1136/hrt.2007.124321 17569811 PMC1994451

[B35] Antman EM Hand M Armstrong PW Bates ER Green LA Halasyamani LK 2007 Focused update of the ACC/AHA 2004 guidelines for the management of patients with ST-elevation myocardial infarction Circulation 2008 117 2 296 329 10.1161/CIRCULATIONAHA.107.188209 18071078

[B36] Estep J Aguilar D Diabetes and heart failure in the post-myocardial infarction patient Curr Heart Fail Rep 2006 3 4 164 169 10.1007/s11897-006-0017-7 17129509

[B37] The Long-Term Intervention with Pravastatin in Ischaemic Disease (LIPID) Study Group Prevention of cardiovascular events and death with pravastatin in patients with coronary heart disease and a broad range of initial cholesterol levels N Engl J Med 1998 339 19 1349 1357 9841303 10.1056/NEJM199811053391902

[B38] Snow V Aronson MD Hornbake ER Mottur-Pilson C Weiss KB Lipid control in the management of type 2 diabetes mellitus: a clinical practice guideline from the American college of physicians Ann Intern Med 2004 140 8 644 649 10.7326/0003-4819-140-8-200404200-00012 15096336

[B39] MRC/BHF heart protection study of cholesterol-lowering with simvastatin in 5963 people with diabetes: a randomised placebo-controlled trial Lancet 2003 361 9374 2005 2016 12814710 10.1016/s0140-6736(03)13636-7

[B40] Baigent CKA Kearney PM Efficacy and safety of cholesterol-lowering treatment: prospective meta-analysis of data from 90.056 participants in 14 randomised trials of statins Lancet 2005 366 9493 1267 1278 16214597 10.1016/S0140-6736(05)67394-1

[B41] Mukherjee D Fang J Chetcuti S Moscucci M Kline-Rogers E Eagle KA Impact of combination evidence-based medical therapy on mortality in patients with acute coronary syndromes Circulation 2004 109 6 745 749 10.1161/01.CIR.0000112577.69066.CB 14970110

[B42] Gaede P Vedel P Larsen N Jensen GVH Parving H-H Pedersen O Multifactorial intervention and cardiovascular disease in patients with type 2 diabetes N Engl J Med 2003 348 5 383 393 10.1056/NEJMoa021778 12556541

[B43] Grant RW Meigs JB Overcoming barriers to evidence-based diabetes care Curr Diabetes Rev 2006 2 261 269 10.2174/157339906776818604 18220631

[B44] Naderi SH Bestwick JP Wald DS Adherence to drugs that prevent cardiovascular disease: meta-analysis on 376,162 patients Am J Med 2012 125 9 882 887 e1 10.1016/j.amjmed.2011.12.013 22748400

[B45] Sanfelix-Gimeno G Peiro S Ferreros I Perez-Vicente R Librero J Catala-Lopez F Adherence to evidence-based therapies after acute coronary syndrome: a retrospective population-based cohort study linking hospital, outpatient, and pharmacy health information systems in Valencia, Spain J Manag Care Pharm 2013 19 3 247 257 Epub 2013/03/30 23537459 10.18553/jmcp.2013.19.3.247 PMC10438044

[B46] Rasmussen S Abildstrom SZ Rasmussen JN Gislason GH Schramm TK Folke F Hospital variation in use of secondary preventive medicine after discharge for first acute myocardial infarction during 1995–2004 Med Care 2008 46 1 70 77 Epub 2007/12/29 10.1097/MLR.0b013e3181484952 18162858

